# Comparative response mechanisms of two cultivars of *Musa paradisiaca L.* to *Fusarium oxysporum* f.sp. *cubense* infection

**DOI:** 10.3389/fpls.2024.1492711

**Published:** 2025-01-09

**Authors:** Yajie Duan, Zhiwei Jia, Zhiwei Lu, Huigang Hu, Rulin Zhan

**Affiliations:** ^1^ School of Tropical Agriculture and Forestry, Hainan University, Haikou, China; ^2^ Key Laboratory of Tropical Fruit Biology, Ministry of Agriculture and Rural Affairs, Key Laboratory of Hainan Province for Postharvest Physiology and Technology of Tropical Horticultural Products, South Subtropical Crops Research Institute, Chinese Academy of Tropical Agricultural Sciences, Zhanjiang, China; ^3^ Fruit Tree Center, Tropical Crops Genetic Resources Institute of Chinese Academy of Tropical Agricultural Sciences, Haikou, China

**Keywords:** *Musa paradisiaca L.*, *Fusarium oxysporum*, disease resistance mechanism, differentially expressed gene, ligin

## Abstract

With the aim of enhancing plants’ ability to respond to pathogenic fungi, this study focuses on disease resistance genes. We commenced a series of investigations by capitalizing on the pronounced differences in resistance to Fusarium wilt between resistant and susceptible varieties. Through an in-depth exploration of the metabolic pathways that bolster this defense, we identified genes associated with resistance to *Fusarium oxysporum* f. sp. *cubense* (Foc). For our analysis, root tissues from seedlings that had been in contact with *Fusarium oxysporum* for four days were harvested, including both infected and uninfected samples, which served as our study specimens. The crude extract treatment led to a significant increase in malondialdehyde (MDA) levels, lignin content, and phenylalanine ammonia lyase (PAL) activity. Conversely, there was a notable decline in protein content, ergosterol levels, and pectinase activity. In the control group, it was observed that 4,474 genes in the resistant varieties were significantly up-regulated compared to the susceptible varieties. The functional annotation of these differentially expressed genes (DEGs) emphasized their predominant participation in biological processes. Further analysis via the KEGG database revealed that 14 DEGs in the susceptible varieties were particularly enriched in pathways related to plant hormone signaling. Through the perspective of transcriptome data, we focused on genes associated with lignin and cell wall development for Q-PCR validation. Notably, the expression levels of Macma4_02_g07840 (COMT) and Macma4_10_g06530 (CCOAOMT) were relatively elevated. Our findings suggest that the resistance of these varieties to wilt infection can be ascribed to the accumulation of lignin metabolites, which inhibits pathogenic fungus growth by restricting the synthesis of cellular metabolites. The evidence documented in our research provides a framework for a deeper understanding of the disease resistance mechanisms in bananas, laying a solid theoretical foundation for future studies in this area.

## Highlights

- After inoculation with Foc4, 4,474 genes in the resistant *Musa paradisiaca L.* were up-regulated.
- The lignin synthesis pathway plays a key role in banana’s defense against *Fusarium oxysporum*;
- The resistance mechanism of banana to Fusarium wilt was revealed from the physiological level;

## 1 Introduction

Aras and Endes (2023) assert that *Fusarium oxysporum* f. sp. *cubense* (Foc) is a catastrophic soil-borne pathogen that obliterates vascular bundles and eradicates banana plants, positioning it among the most severe fungal infections worldwide. A devastating soil-borne disease that destroys vascular bundles and kills banana plants, ranking among the most serious fungal diseases globally. *Fusarium oxysporum* can continuously produce new physiological strains, and its posterior wall spores persist in the soil, making Fusarium blight challenging to control (Chang et al., 2021; Gilardi et al., 2021). This disease has severely hampered the development of the banana industry. Specialized strains have been detected in numerous banana crops, inflicting substantial damage on their growth and curtailing yields. *Fusarium oxysporum* from different banana crops synthesizes different levels of toxins. The most common toxin is fusaric acid (FA), a toxin with broad-spectrum toxicity that reduces the vitality of banana plant cells and inhibits the activity of defense enzymes (Lv et al., 2020; Jia et al., 2023). At present, the main control measures for banana wilt include grafting, crop rotation and fungicides. However, these methods are costly and cumbersome, readily leading to an increase in pesticide residues and enhanced fungal resistance (Mwangi et al., 2021; Man et al., 2022; Van Westerhoven et al., 2022). Therefore, breeding resistant varieties is the fundamental way to address banana wilt.

Lignin, an aromatic polymer resulting from the oxidative coupling of 4-hydroxyphenylpropane, and its metabolism are crucial for plant growth and development, serving as a significant barrier against pests and many adverse situations (Man et al., 2022; Van Westerhoven et al., 2022). Additionally, lignin is involved in the biological process of plant resistance (Wei et al., 2024). The lignin synthesis pathway is a branch of the plant phenylpropanoid synthesis pathway. Some enzymes involved in lignin synthesis cross with other enzymes involved in phenylpropane synthesis (Ahmed et al., 2021; Abiola et al., 2024). Therefore, lignin synthesis is related to the synthesis of other phenylpropanoid substances in plants under adverse conditions such as disease, waterlogging, and low temperature (Jia et al., 2023). Mwangi et al. reported that the total phenolic content and the flavonoid content of pisolin and phaseolin in tobacco resistant to red starpox were significantly greater than those in susceptible varieties (Mwangi et al., 2021). Zhou et al. reported resistance to verticillium wilt in grafted eggplants and provided evidence that in the non-infected state, PAL activity, phenolic substance content, and lignin content in the plants were significantly greater than those in the control plants overall, and the increase in these two indices was even more pronounced in control plants after infection (Zhou et al., 2023). To some extent, PAL activity and phenolic substance content are correlated with verticillium wilt resistance in eggplant. In buckwheat seedlings and different tissues, the flavonoid content initially increased and then decreased with increasing PAL activity, following a chronological sequence. These findings indicate that non-lignin metabolism and lignin metabolisms, involving phenylpropane substances, are associated with disease and stress resistances in plants (Zhu et al., 2023).

The development of plant disease resistance is an extremely complex process and changes in physiological indicators such as photosynthetic capacity, the antioxidant system and osmoregulatory substances involve the expression of many genes and the regulation of signaling pathways (Ahmed et al., 2021; Abiola et al., 2024). Exploring the important resistance genes associated with different resistance levels under fusarium wilt stress and elucidating the signal transduction pathway and regulatory network are crucial for understanding the molecular mechanism of fusarium wilt resistance in bananas (Assis Ferreira Gadelha et al., 2021; Bhatti et al., 2022). Lignin is an organic macromolecule that is second only to fibrotin and plays a significant role in plant mechanical support, water transport, and resistance to external microorganisms. In this study, we investigated the epigenetic properties of key enzymes in the synthesis of lignin in different banana varieties and under the treatment of Foc4, using the methods of plant biology and merophysiology. The transcriptional and regulatory factors of the lignin synthesizing key factor *C3H* were determined, and the synthesis characteristics of banana lignin were studied. In our study, transcriptome sequencing analysis was performed on two varieties of *Musa paradisiaca L*. (disease-resistant and susceptible) after inoculation with Fusarium, and the differentially expressed genes and regulatory pathways of the two varieties were combined to predict the resistance response mechanism of the two varieties of *Musa paradisiaca L*. These differences were compared, laying a foundation for the study of the disease resistance mechanisms of *Musa paradisiaca L*.

## 2 Materials and methods

### 2.1 Fusarium strains, medium cultivation and disease index standard

The experimental strain used was *Fusarium oxysporum* f. sp. cubense variety 4 (Foc4) with green fluorescence (GFP), an organism that has long been applied experimentally and has the same virulence as the wild-type strain without GFP. The experimental banana seedlings (Jin Long and Jinsha Xiang) were plants derived from tissue culture, with 5 leaves and a height of approximately 15 cm. The seedlings were planted in pots with a diameter of 10 cm in nutrient-enriched soil. Twenty-five banana seedlings with good growth conditions and relatively consistent growth were selected for each variety for subsequent inoculation with Foc4. The disease-resistant varieties of Jin Long were labelled X1, and the susceptible varieties of Jinsha Xiang were labeled J. All the experimental materials were provided by the Institute of South Subtropical Crops, Chinese Academy of Tropical Agricultural Sciences.

The media utilized for testing were PDA solid medium and YPD liquid medium. Two hundred grams of peeled potatoes were diced, combined with 800 ml of distilled water, boiled in an induction cooker for 12 minutes, and subsequently filtered using double gauze. Subsequently, 20 g of anhydrous glucose was added to the filtrate, stirred until dissolved, and the volume was adjusted to 1 L with distilled water. The YPD liquid medium consisted of 10 g/L yeast extract, 20 g/L tryptone, 20 g/L glucose, and 1 L of distilled water (Devika and Saha, 2024) (Devika and Saha, 2024).

Seeds of disease-resistant and disease-susceptible *Musa paradisiaca L*. were selected and cultured in an incubator at 25 ± 2℃ with a light intensity of 5000 lx for 12 h. Two groups were established: a treatment group consisting of Jin Long banana seedlings and a control group with the pathogenic bacteria of Jinsha Xiang banana seedlings. Each group had three replicates, and each replicate contained ten seedlings. When the seedlings developed two leaves, the treatment group was inoculated with a *Fusarium oxysporum* spore suspension (concentration: 1 × 10^8^/mL) using the root injury or root immersion method for 1 h. The disease was classified into the following grades: Grade 0, asymptomatic; Grade 1, cotyledon yellowing without wilting; Grade 2, cotyledon wilting or mild plant wilting; Grade 3, obvious plant wilting or dwarfing; Grade 4, dead plants. The control group was treated with an equal amount of sterile water, and the first true leaf of the control seedlings inoculated with fusarium wilt for 2 days was preserved in liquid nitrogen (George-Opuda et al., 2023).

### 2.2 Microscopic observation of the infection process

Fresh root samples of disease-resistant (X1) plants from 12–360 h after inoculation were sliced by hand and observed under a confocal laser microscope. The sterile water inoculation treatment (J) was used as the control. Simultaneously, fresh root samples of X1 plants were inoculated with sterile water 12−168 h after inoculation the plants were cleaned with sterile water, immediately frozen with liquid nitrogen for more than 15 min, and then stored in an ultralow-temperature refrigerator at -80°C until use. Three varieties of banana roots inoculated with GFP-Foc4 were sliced manually and temporary slides were future made. Foc4 infection in different parts of different varieties of bananas was observed via confocal laser microscopy and bright field imaging. The wavelength of excitation light for GFP green fluorescence observation was 488 nm. The wavelength of the emitted light was 520 nm, the wavelength of the spontaneous fluorescence (red fluorescence channel) excitation light of the banana plant tissue was 543 nm, and the wavelength of the emitted light was 590 nm (Kok and Irawati, 2020).

### 2.3 Effects of antagonistic bacteria on the related indices of pathogens

The effects of antagonistic bacteria on the lipid peroxidation and ergosterol levels of pathogenic fungi and the protein content and pectinase activity of pathogenic mycelia were investigated using the corresponding kit (purchased from Tiangen Biochemical Technology (Beijing) Co., Ltd.).

The dried mycelia were ground into a powder using the saponification method and 0.10 g was weighed and added to a mixture of 10 mL of methanol and chloroform (volume ratio of 3:1). After mixing, the mixture was extracted with ultrasonication three times for 15 min each. The extraction mixture was centrifuged at 3000 rpm for 5 min and the supernatant was removed, followed by the addition of 10 mL each of water, chloroform, and 0.5 mol/L potassium phosphate buffer containing 2.0 mol/L potassium chloride (pH 6.8). After extraction and stratification the chloroform phase was removed and rotated to dryness at 45°C. A mixture of methanol and ethanol (volume ratio of 4:1) containing 1.4 mol/L potassium hydroxide was added to a final concentration of 10 mL and saponified at 60°C for 1.0 h. After adding 10 mL of water and 10 mL of petroleum ether the extracted phase of petroleum ether was distilled at 37°C, the volume of ethanol was fixed to 10 mL, and the mixture filtered through a microporous organic filter membrane (pore size, 0.22 μm). Detection was performed using the UPLC external standard method, and each treatment was repeated three times (Méndez-Cantillo et al., 2022; Macedo Viana et al., 2022).

### 2.4 Transcriptome sequencing and differential expression gene analysis of *Musa paradisiaca L.*

An RNAprep Pure Plant Kit was used to extract total RNA from the leaves, a Nanodrop and GX were used to determine the purity, concentration and integrity of the RNA, and a cDNA library was constructed after the quality inspection was completed. The Illumina HiSeq 2500 platform was used for high-throughput sequencing after the construction of the database.

The transcriptome data were screened for differentially expressed genes with read contamination detection and quality control, *de novo* splicing and statistical analysis, and unigene expression annotation. With (p2) as the screening criterion, the expression abundance of each unigene was calculated via the Bowtie2 method and the express method via the splicing unigene database. Differential genes were screened via the FPKM method. The screening criteria for differentially expressed genes were a false recovery rate ≤ 0.001 and a |log2(fold change)| ≥ 1. Differentially expressed genes were compared to data in the GO and KEGG databases and functional enrichment analysis was performed among these differentially expressed genes. Through significance analysis of the data, metabolic pathways significantly enriched in the DEGs were identified. For the metabolic pathways identified in this study, p values ≤ 0.05 were considered significantly enriched DEG pathway. Pathway analysis of the DEGs allowed for the screening of resistance-related metabolic pathways and key DEGs (Vilhena et al., 2020; Ordonez-Santos and Garzon-Garcia, 2021).

### 2.5 Fluorescent quantitative PCR to identify key differentially expressed genes

According to the GO and KEGG annotation results, five genes were selected and their gene expression levels were verified with qRT−PCR. With Actin as the internal reference gene ( (He and Brakebusch, 2024; Shen and Jiang, 2024; Sundby et al., 2024), the products were amplified with qPCR using a LightCycler 96 Real-Time System (Roche, Switzerland). The product length was between 70 and 128 bp, the Tm value was 60°C, the GC content was 55%, and each sample was analyzed three times. The relative expression of the DEGs was calculated using the 2^−ΔΔCT^ method (Owusu-Boadi et al., 2021; Oyeyinka and Afolayan, 2021).

### 2.6 Statistical analyses

The data sets were replicated more than three times, all data were exported and processed by the Microsoft Excel program and were analyzed using the IBM SPSS version 22. All data were presented as mean values with their standard deviations (mean ± standard deviation, *n* = 8). At a 95% confidence level, ANOVA and LSD multiple comparison tests were used for statistical analyses. ^*^ *P* < 0.05 and ^**^ *P* < 0.01 indicated statistically significant differences.

## 3 Results

### 3.1 Observation of strain morphology

In this experiment, the *Fusarium oxysporum* f. sp. *cubense* variety 4 (Foc4) strain with green fluorescence (GFP), which has been utilized in experiments over an extended period and exhibits the same virulence as the wild-type strain, was employed. The activated fungus mixture of Foc4 in the YPD liquid medium was carefully shaken and diluted on a super clean workbench to prepare temporary slides for the observation of strain morphology and fluorescence intensity. It was observed that Foc4 grows vigorously, and bright fluorescence can be clearly seen in both spores and mycelia. These characteristics enable its potential use in subsequent processes of infecting and colonizing banana plants.

**Figure 1:**
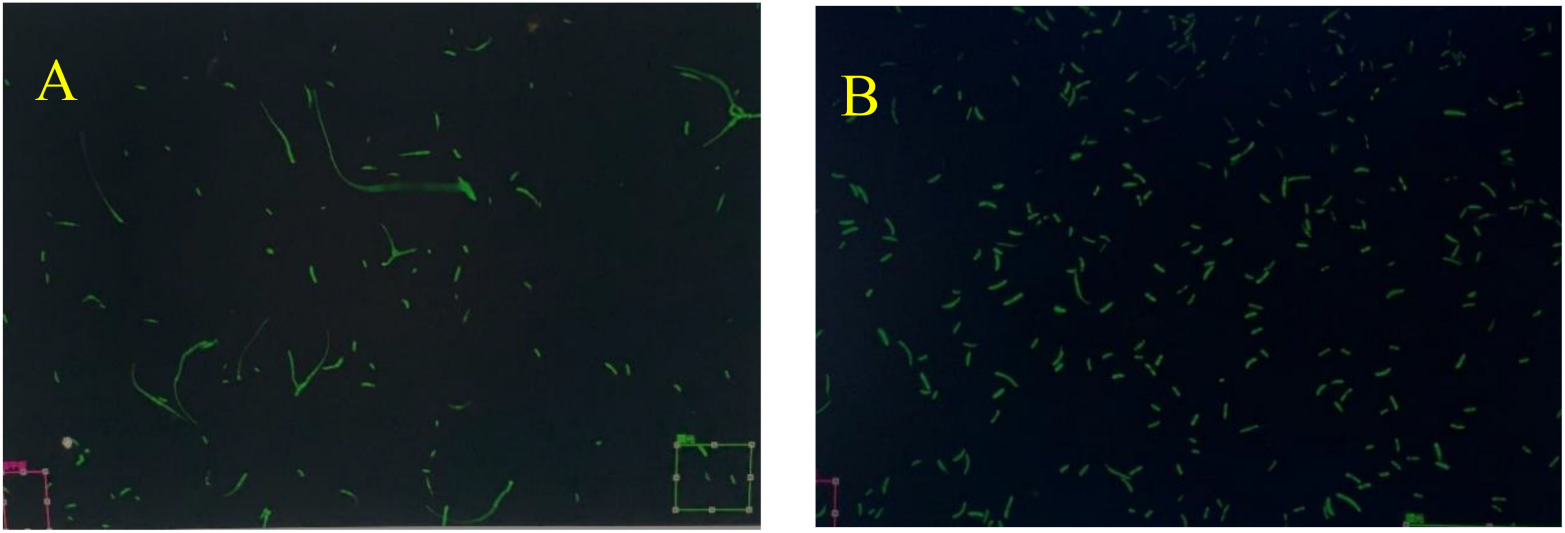
Observation of Strain Mycelium and Spore Morphology. GFP = Green Fluorescent Protein; Foc TR4 = *Fusarium oxysporum* f. sp. *cubense* race 4; X1 = disease-resistant variety ‘Jin Long’; J = susceptible variety ‘Jinsha Xiang’. This applies to the subsequent figures. **(A)** is the mycelium morphology of *Fusarium oxysporum* f. sp. *cubense* race 4 (Foc4) after ten subcultures, and **(B)** is the spore morphology of the same after ten subcultures.

The mixture was processed with precision to ensure the quality of the temporary slides. Subsequently, through observation using a confocal laser microscope, it was determined that neither the spore morphology nor the size had undergone any alteration. Additionally, pathogenicity tests indicated that the virulence of the strain had not weakened (**Figure 1**).

### 3.2 Histopathological observation of different varieties of bananas infected with Foc4

**Figure 2:**
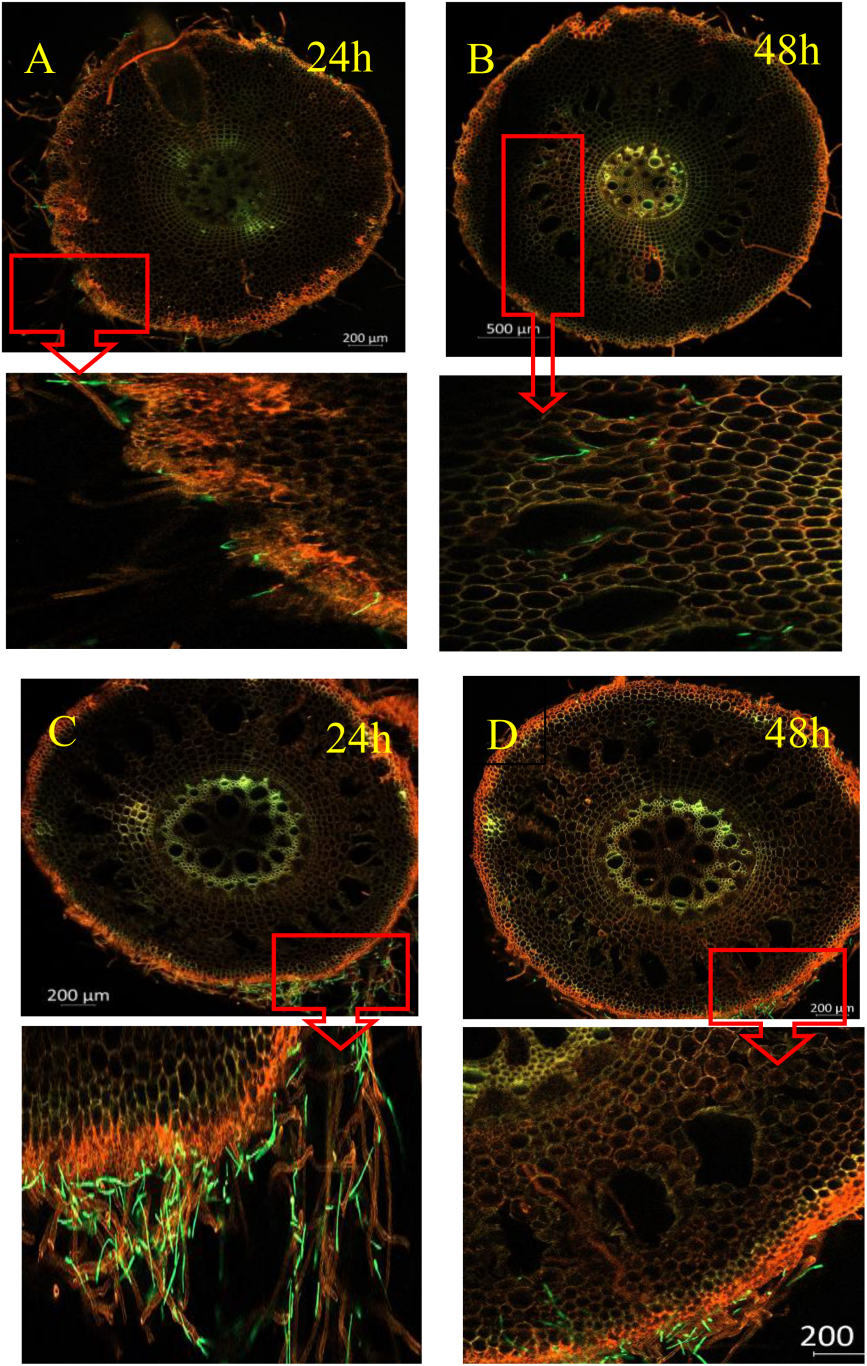
Spatial and Temporal Distribution of GFP - Foc TR4 in Different Varieties After Inoculation (X1 and J). The figure shows the infection status of GFP - Foc TR4 in the root systems of banana varieties X1 (resistant) and J (susceptible) at different times (24h, 48h, etc.). The green fluorescence indicates GFP - Foc TR4. The pathogen’s infection progress can be inferred by observing the green signal’s distribution and intensity in the roots. Magnified images **(A–D)** are provided below for detailed viewing.

As shown in **Figure 2**, Foc4 spores and mycelia attached to the epidermal cells of the root 24 h after infection. Subsequently, at 48 h, they penetrated the root cortical cells. However, the number of spores and mycelia that passed through differed, with X1 having significantly fewer spores than J, suggesting that the cortical cells of X1 may have a certain blocking effect on Foc4 (**Figures 2C, D**). At 48 h, the Foc4 mycelium started to expand longitudinally along the intercellular space and entered the xylem duct, attaching to the cell wall and intercellular space. By 168 h after infection, the peripheral vascular bundle system began to collapse, which was only observed in J plants (**Figures 2A, B**).

### 3.3 Determination of antioxidant defense enzyme system activity

**Figure 3:**
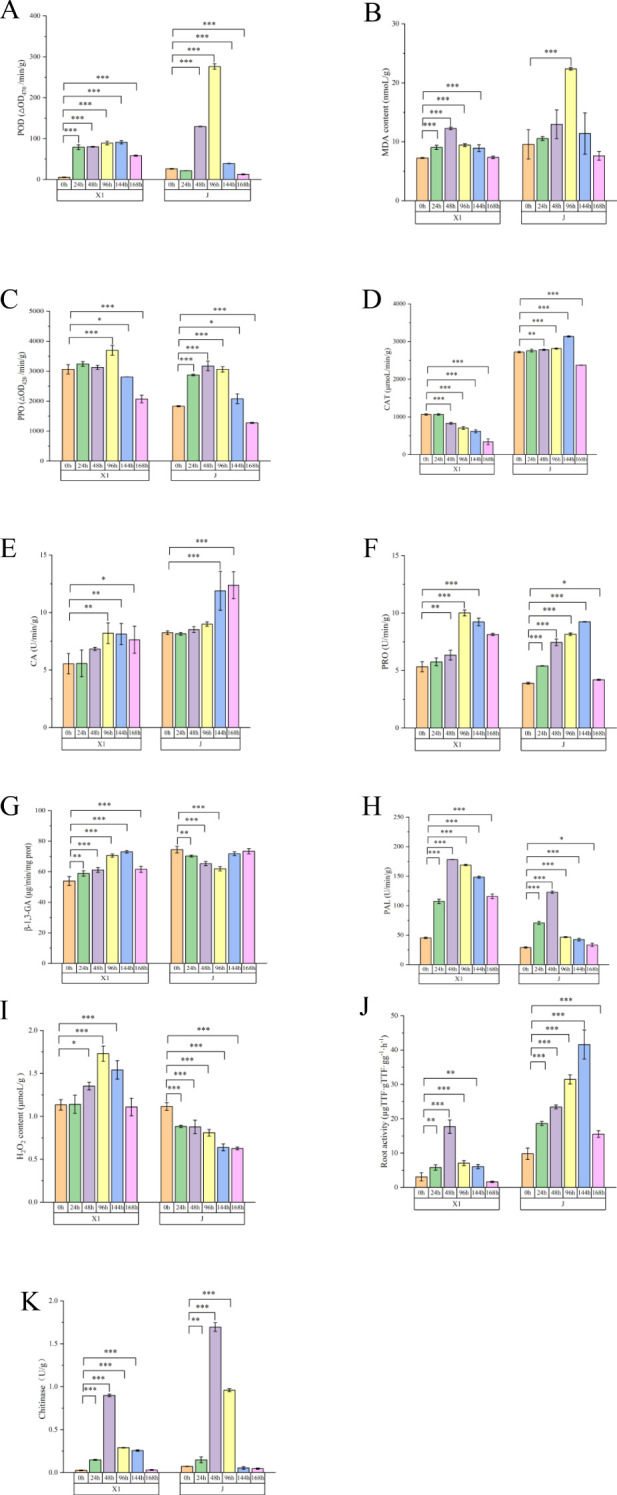
Analysis of the Physiological Indices of *Musa paradisiaca L.* The horizontal axis represents different varieties at different time points after inoculation (24h, 48h, 96h), and the vertical axis represents the expression levels of different physiological data. **(A)** Peroxidase (POD) content (ΔOD_470_/min/g); **(B)** Malondialdehyde (MDA) content (nmol/g); **(C)** Polyphenol Oxidase (PPO) content (ΔOD_420_/min/g); **(D)** Catalase (CAT) content (μmoL/min/g); **(E)** Carbonic Anhydrase(CA) content (mg/g); **(F)** Proline(PRO) content (μg/g); **(G)** β-1,3-Glucanase (β-1,3-GA) content (μg/min/mg prot); **(H)** Phenylalanine Ammonia-Lyase (PAL) content (ΔOD_290_/h/g); **(I)** Hydrogen Peroxide (H_2_O_2_) content (μmol/g); **(J)** Root activity (μg/h/g); **(K)** Chitinase content (U/g).Data are mean ± standard deviation, *n* = 8.*P < 0.05, **P < 0.01,***P < 0.001 (compared to 0 h, two-way ANOVA corrected by Sidak’s multiple comparisons test).

The activities of defense enzymes in the root samples of the resistant varieties inoculated with Foc TR4 for different periods were determined. These enzymes mainly included those related to lignin and phenolic compounds, such as lignin content, phenylalanine aminolyase (PAL) activity, malondialdehyde (MDA) content, total flavonoids, total phenol (TP) content, and polyphenol oxidase (PPO) activity. Notably, during the 24 h - 48 h period, the lignin content of the resistant variety X1 showed a significant difference compared to that of the susceptible variety. In contrast, no differences were detected in chitinase activity, β-1,3-glucanase activity, H_2_O_2_ concentration, peroxidase dismutase activity, or peroxidase activity. Generally, changes or accumulations of defense enzymes associated with resistance can inhibit or delay Foc TR4 infection to some extent, which is consistent with the results of histological observation. The differences among the resistant varieties were mainly related to the synthesis pathway of phenylalanine and lignin (**Figure 3**).

### 3.4 Phenotypic observation of *Musa paradisiaca L.* after inoculation

**Figure 4:**
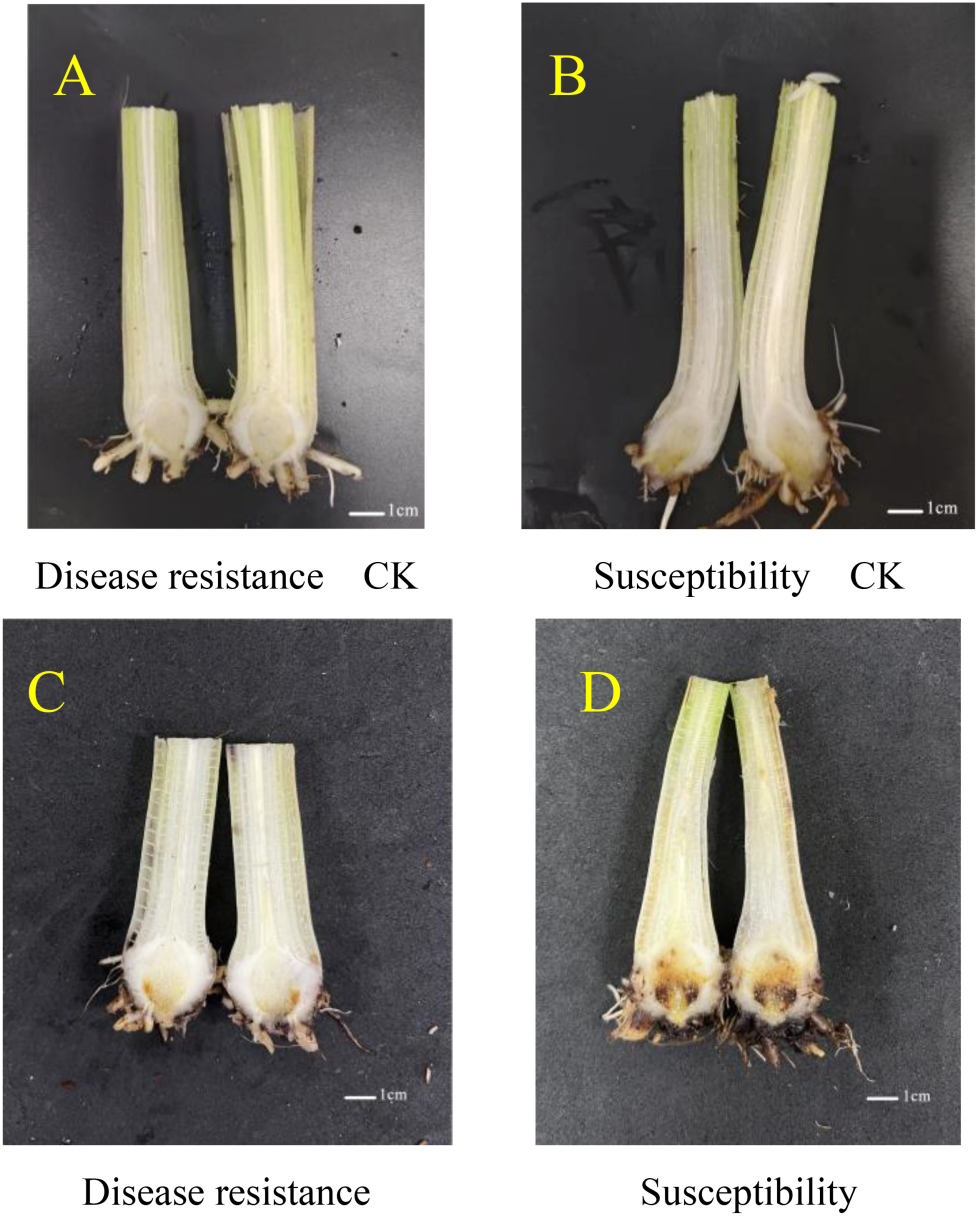
Wilt Conditions of *Musa paradisiaca L*. Seedlings Before and After Inoculation with *Fusarium oxysporum.* **(A, B)** respectively depict the growth states of the banana seedlings of the disease-resistant variety X1 and the susceptible variety J prior to inoculation, serving as normal controls. **(C, D)** display the conditions at 50 days post-inoculation. In the case of the X1 variety **(C)**, the root browning and disease severity are relatively mild, with partial root discoloration being observed and the overall growth of the plant being minimally affected. In contrast, for the J variety **(D)**, extensive root browning is evident, the vascular bundle system is largely disrupted, and the plant exhibits pronounced wilting or stunting, thus manifesting severe disease symptoms.

When the seedlings produced two leaves, they were inoculated with a *Fusarium oxysporum* suspension at a concentration of 1 × 10^8^/mL. Prior to inoculation, the growth conditions of the banana seedlings of the disease-resistant variety X1 and the susceptible variety J were documented as the normal controls. After a 50-day post-inoculation period, it was observed that in the X1 variety, the browning of the roots and the extent of disease development were relatively moderate, characterized by a minor degree of discoloration in a portion of the roots and a comparatively negligible impact on the overall growth of the plant. Conversely, in the J variety, a large area of root browning was apparent, the vascular bundle system was substantially damaged, and the plant exhibited significant wilting or stunting, thereby presenting severe symptoms of the disease (**Figure 4**).

### 3.5 Analysis of differentially expressed genes

**Figure 5:**
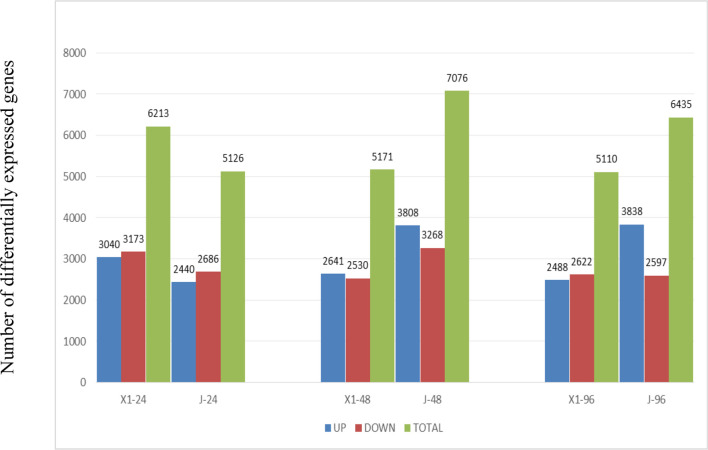
The number of DEGs in Two varieties *Musa paradisiaca L.* the x-axis represents the time after inoculation (24h, 48h, 96h), and y-axis represents the number of differentially expressed genes. The blue bar graph represents the number of differentially expressed genes in the disease-resistant variety X1 at different time points, and the red bar graph represents the corresponding situation of the susceptible variety J.

After 4 days of treatment, the differentially expressed genes (DEGs) of the two varieties were compared with those related to GO entries (**Figure 5**). The results showed that at 4 days after inoculation, the DEGs were abundant. In terms of GO categories, the molecular function was secondary, and the cell component had the least enriched genes. Among the biological processes, single-organism processes, intracellular processes, and metabolic processes had the most enriched genes. In the molecular function category, binding, catalytic activity, and molecular function were the most enriched functions. For cell components, cells, cell components, membranes, membrane components, and organelles had the most abundant genes.

### 3.6 GO enrichment analysis of the DEGs of the two *Musa paradisiaca L.* varieties

In the GO enrichment analysis, among the significantly enriched GO terms in susceptible *Musa paradisiaca L.*, 57 belonged to the molecular function category, of which 29 were related to enzyme activity and 20 were related to binding. The remaining 8 terms included the structural composition of ribosome (GO: 0003735), molecular structure-activity (GO: 0005198), and other activities related to transmembrane transporters (such as nuclear base, xanthine, and purine nuclear base transporters) and ATP hydrolysis (GO: 0016887), as well as protein dimerization activity (GO: 0046983). Thirty-one items were cell components (CCs) including organelles and organelle components: such as cell wall (GO: 0005618), ribosome (GO: 0005840), organelle (GO: 0043226), plasmodesma (GO: 0009506), nucleosome (GO: 0000786), and mitochondria (GO: 0000786), and also polymerized cytoskeletal fibers (GO: 0099513), supramolecular polymers (GO: 0099081), driver protein complexes (GO: 0005871), and DNA packaging complexes (GO: 0044815). The 50 biological processes involved in cell wall and lignin synthesis included cell wall organization (GO: 0071555), microtubule formation (GO: 0007017), pectin metabolism (GO: 0045488), and oxidative stress (GO: 0045488), flavonoid biosynthesis (GO: 0009813), cell wall polysaccharide metabolism (GO: 0010383), cell wall modification (GO: 0042545), and other significantly expressed processes.

Similarly among the significantly enriched gene ontology (GO) terms in disease-resistant *Musa paradisiaca* L., 29 of the significantly enriched. GO terms pertained to molecular functions. Among these genes, 16 were associated with enzyme activities, uch as ubiquitin - protein transferase activity (GO: 0010383), oxidoreductase activity (GO: 0016705), and growth factor activity (GO: 0008083), and these activities are linked to potent antioxidant properties. Furthermore, 9 of these categories were related to binding activities, primarily involving binding to iron ions (GO:005506) and acetylcholine receptors (GO: 0033130).

**Figure 6:**
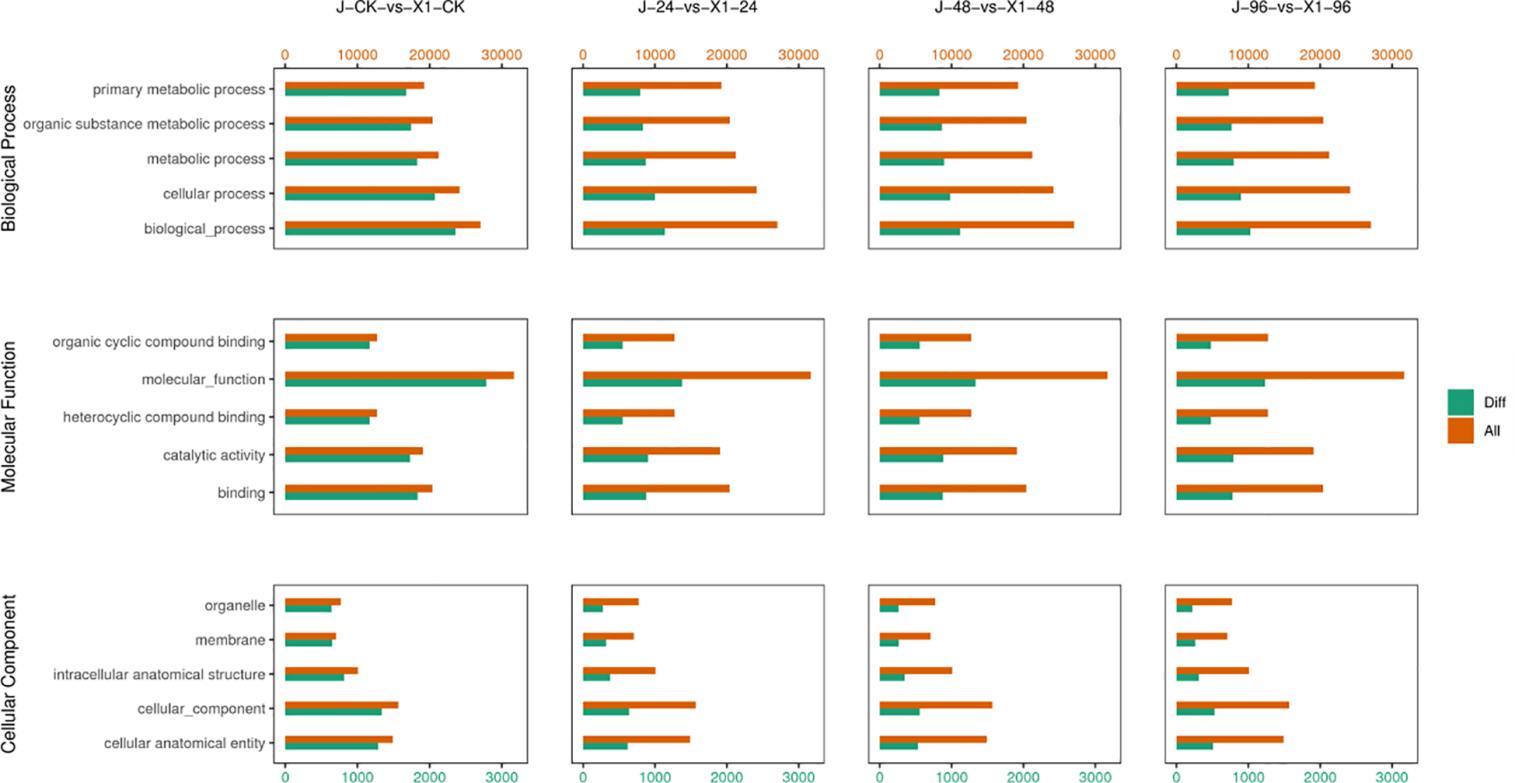
Comparison of the Differences in the GO functional Classification of DEGs between Two Varieties *Musa paradisiaca L.* the horizontal axis represents the GO categories, covering aspects including molecular function, cellular component, and biological process. The vertical axis represents the degree of enrichment (usually indicated by indicators such as Enrichment Factor or the number of enriched genes). Bar graphs or scatter plots of different colors (selected based on specific circumstances) represent the gene enrichment of different varieties.

After the initial impact of *F. oxysporum*, 51 terms related to biological processes, including protein phosphorylation (GO: 0006468), ubiquitin-dependent protein degradation (GO: 0043161), regulation of cellular biosynthesis (GO: 0031326), and regulation of macromolecule metabolic processes (GO: 0010556), were identified. Apparently, the primary response to this impact was the activation of a substantial number of repair-related biological processes, which is illustrated in **Figure 6**.

### 3.7 KEGG metabolic pathway analysis of differentially expressed genes

**Figure 7:**
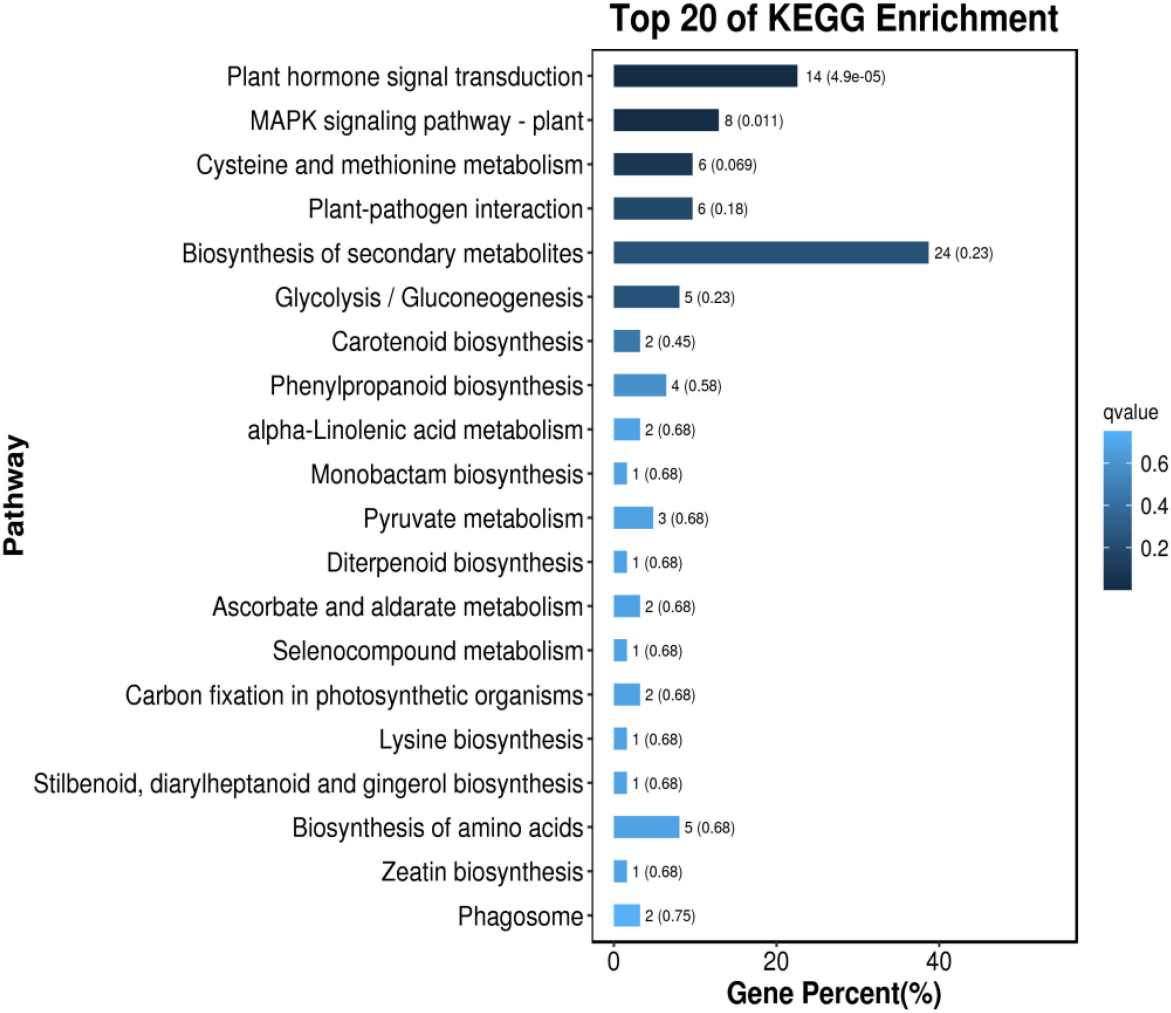
Comparison of the Top 20 Most Enriched KEGG Pathways in Different Varieties of *Musa paradisiaca L*. the horizontal axis represents the number of genes annotated in each KEGG metabolic pathway, and the vertical axis represents the names of the KEGG metabolic pathways. The size of the bubble represents the number of genes enriched in the pathway. The larger the bubble, the more differentially expressed genes are involved in the pathway.

KEGG pathway analysis was conducted on the DEGs in Foc4 following a 24-h infection period (Macedo Viana et al., 2022; Widoyanti et al., 2023). As depicted in **Figure 7**, genes expressed differently in resistant varieties were notably enriched in several pathways including plant hormone signal transduction (ko04075), biosynthesis of flavonoids and flavonols (ko00944), phenylpropanoid biosynthesis (ko00940), flavonoid biosynthesis (ko00941), and the biosynthesis of various secondary plant metabolites (ko00999). Upon infection by the pathogen the genes that exhibited differential expression predominantly focused on pathways such as the ribosome (ko03010), cytophagosome (ko04145), carbon metabolism (ko01200), biosynthesis of secondary metabolites (ko01110), the TCA cycle (ko00020), starch and sugar metabolism (ko00500), and carbon fixation in photosynthetic organisms (ko00710).

### 3.8 Fluorescence quantitative PCR analysis

**Figure 8:**
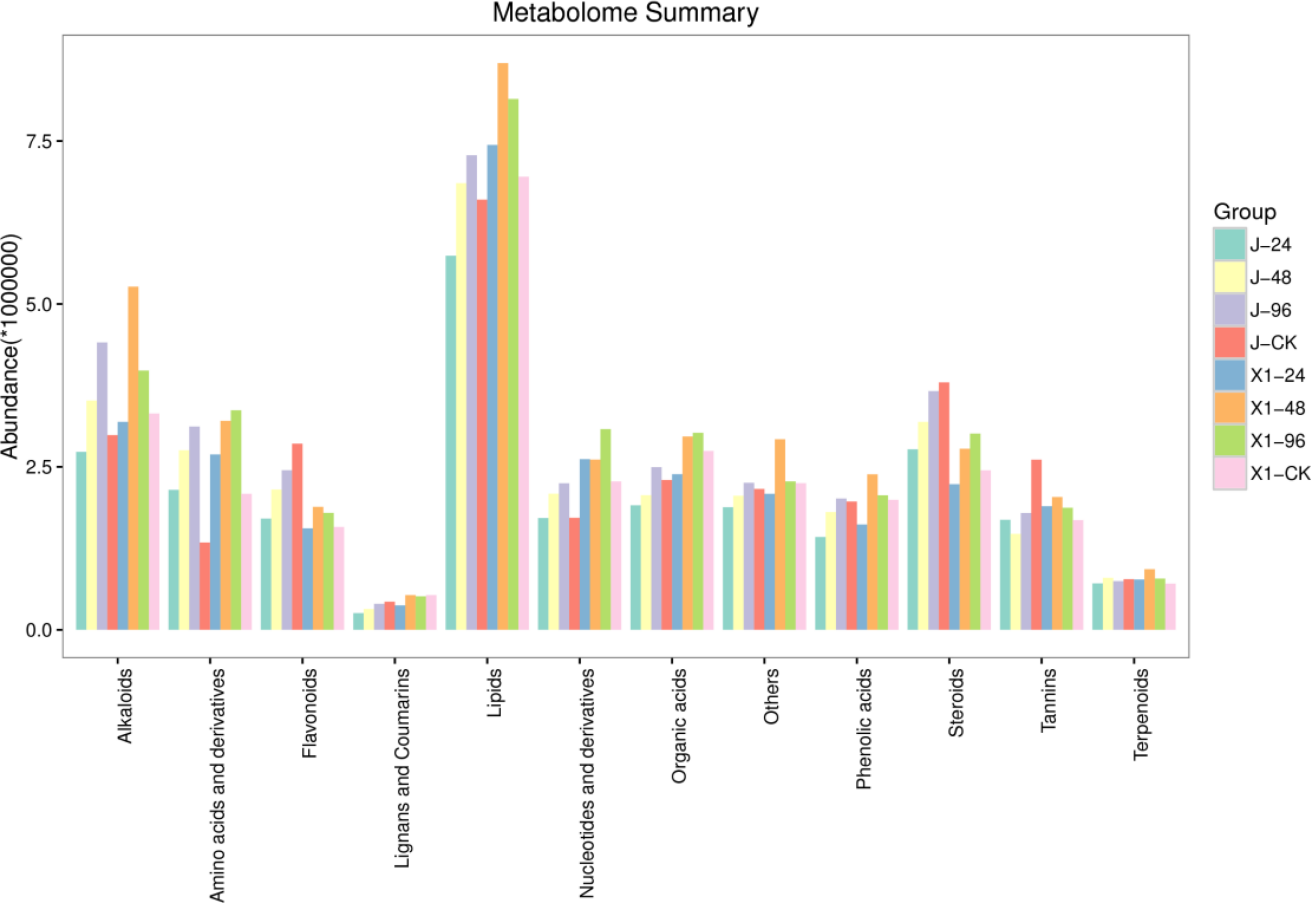
PCR Validation of the common differentially expressed genes in two varieties of *Musa paradisiaca L.* the horizontal axis, colored differently, represents the banana varieties (X1 and J) and the time points after inoculation respectively; the vertical axis presents the relative expression level of genes as the 2^−ΔΔCT^ value. The bar graphs of different colors correspond to different differentially expressed genes.

Differentially expressed genes associated with metabolic pathways related to antagonism were selectively analyzed via quantitative real-time PCR (qRT−PCR). These findings highlighted that the lignin metabolism pathway was enriched predominantly in lipid metabolism and genes belonging to the MYB family. Furthermore, compared with those in susceptible varieties the gene expression levels in resistant varieties were significantly greater and analysis of the metabolome pathways revealed an increase in lipid substances within the cytoplasm of these resistant varieties (**Figure 8**).

### 3.9 MaCCoAOMT is an important gene involved in lignin synthesis in resistant varieties

**Figure 9:**
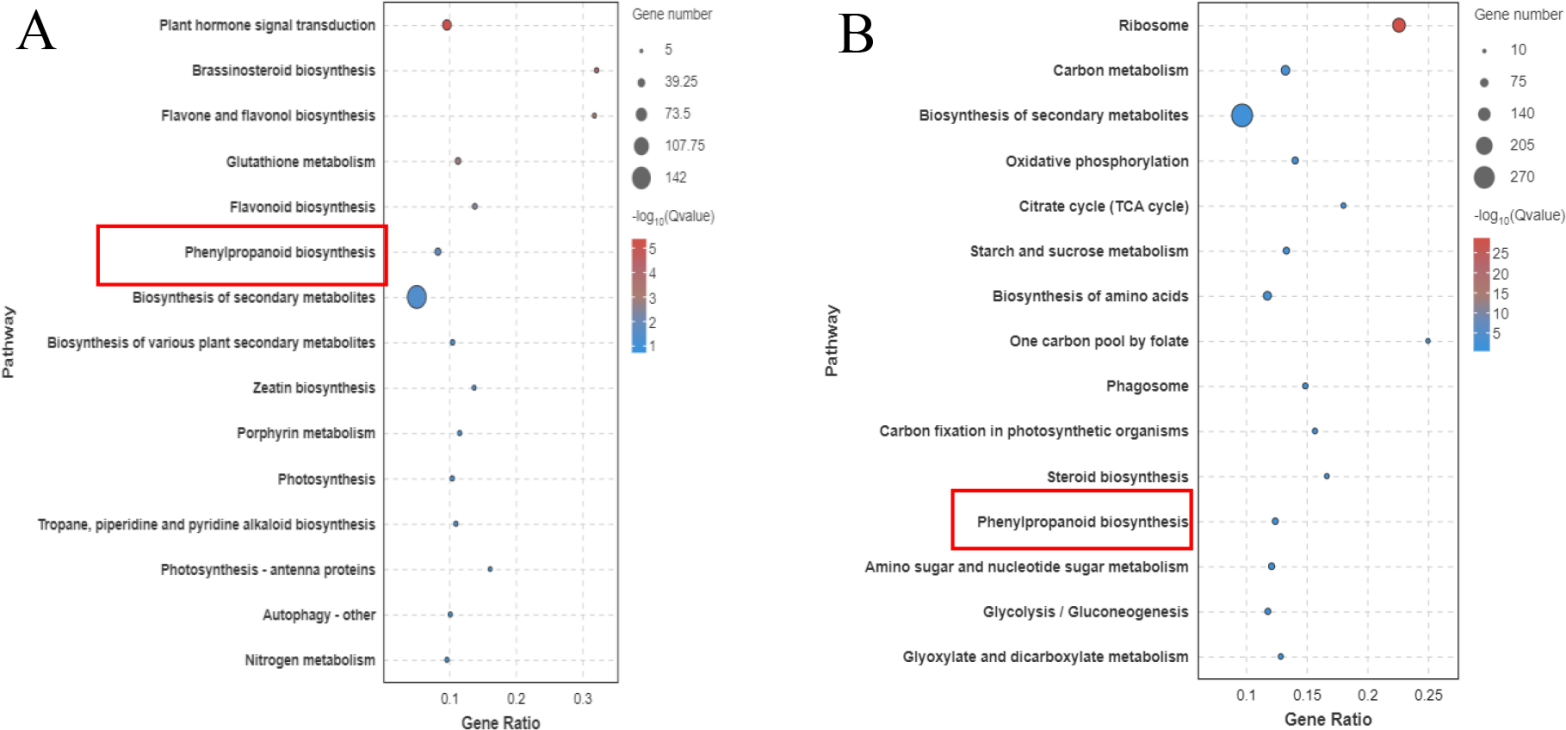
KEGG Enrichment Pathways of Different Banana Varieties after Foc TR4 Treatment (X1 and J). **(A)** shows the enrichment of differentially expressed genes in the KEGG metabolic pathways of the disease-resistant variety X1 after inoculation with Foc TR4, and **(B)** shows the corresponding results of the susceptible variety J. In the figure, the horizontal axis represents the names of the KEGG metabolic pathways, and the vertical axis represents the number of genes enriched in the pathway (expressed by the number of genes).

The transcriptomes of the resistant variety (X1) and susceptible variety (J) after inoculation with Foc TR4 were sequenced in the early stage and the key enzyme phenylalanine aminolysase (PAL) in the resistant variety (X1) activated the linked phenylpropanoid metabolic pathway. The main upregulated genes in the KEGG pathway were enriched in the lignin synthesis pathway and the flavonoid synthesis pathway of the downstream branch pathway. The phenylpropanoid metabolic pathway and lignin-related pathway in the susceptible variety (J) did not increase gene enrichment (**Figure 9**).

**Figure 10:**
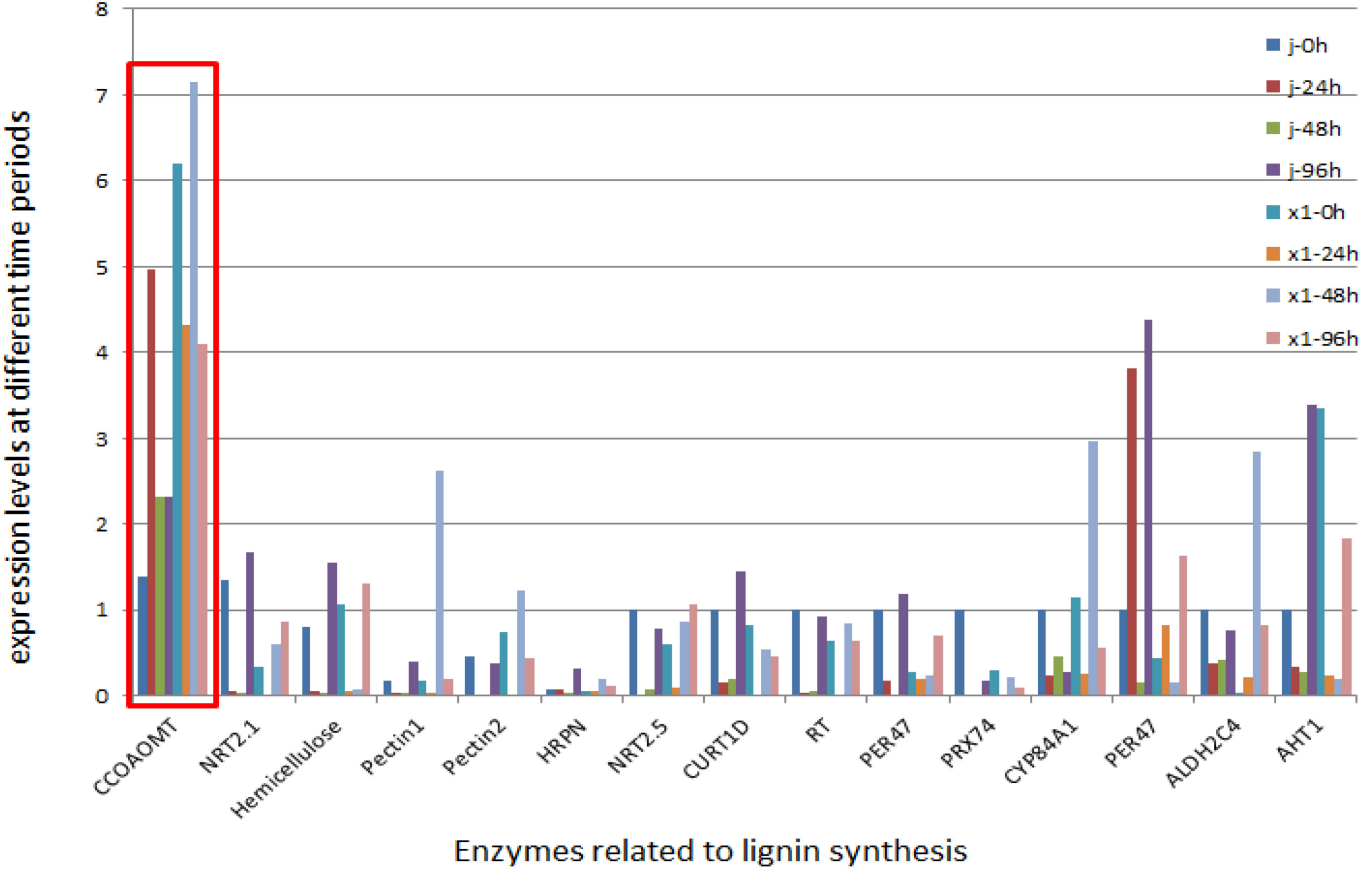
Expression Dynamics of Lignin – Related Differential Genes in Susceptible Banana Varieties after Inoculation. In the figure, the horizontal axis represents the time after inoculation (0h, 24h, 48h, 96h), while the vertical axis represents the relative expression level of genes (which is obtained through specific calculation methods, such as the 2^−ΔΔCT^ value or other normalized expression levels). The lines or bar graphs in different colors stand for different lignin-related differentially expressed genes. Five biological replicates were set at each time point, and the gene expression data was calibrated by the reference gene.

According to the transcriptome data, 2449 DEGs were upregulated in the resistant material. The phenylalanine pathway was more highly expressed in the resistant variety than in the susceptible variety according to the KEGG pathway. Phenylalanine is a front-end reaction of lignin synthesis. Through transcriptome expression analysis, genes related to lignin and the cell wall (*C4H, C3H, CCoAMT, CCR1, COMT, 4CL, PAL, F5H, CAD*) were selected. Through expression analysis, 14 DEGs were screened according to the principles of high expression in resistant varieties and large differential multiples and 83 pairs of primers were designed for these 14 genes to screen for the next experiment. RT−PCR verification of these genes revealed that the expression levels of Macma4_10_g06530 (CCoAOMT) and Macma4_02_g07840 (COMT) were greater and that the expression levels of the disease-resistant varieties were greater than those of the disease-susceptible varieties. Subsequent studies were conducted on these two genes (**Figure 10**).

## 4 Discussion

*Musa paradisiaca L.* is a banana variety possessing numerous excellent genetic traits, including cold tolerance and disease resistance. When infected by fusarium wilt, it exhibits good resistance (Ahmed et al., 2021; Abiola et al., 2024). The threonine metabolic pathway plays a significant role in plant physiological and biochemical regulation. After being attacked by pathogens, the plant hormone signal transduction pathway is activated first. The plant hormones synthesized by this pathway are usually used as signaling factors to activate or regulate the expression of related disease resistance genes. However, most hormones cannot play a direct defense role against pathogens (Chang et al., 2021; Gilardi et al., 2021). In contrast, the disease resistance mechanisms of susceptible bananas may be more direct and effective against the invasion of pathogenic bacteria (Devika and Saha, 2024).

The metabolic pathways of the two varieties were analyzed. In the phenylpropanoid biosynthesis pathway, more upregulated more upregulated differentially expressed genes (DEGs) were concentrated in the resistant varieties compared to the susceptible varieties. These upregulated expressed genes were related mainly to lignin synthesis (Lv et al., 2020; Jia et al., 2023). Lignin is an important physical antifungal substance in plants, and its role in disease resistance has been reported (Ahmed et al., 2021; Abiola et al., 2024). In the interaction between pathogens and host plants the accumulation rate and amount of lignin in resistant varieties were greater than those in susceptible varieties. Moreover, lignin content in tissues increased significantly with increasing plant age. previous studies have provided evidence that the difference between lignin content and disease resistance is highly significant (Vilhena et al., 2020; Jia et al., 2023). The acceleration of lignin synthesis is beneficial for repairing cell wall damage caused by Fusarium wilt infection in host plants. It further lignifies the cell wall, forming a lignified barrier that prevents Fusarium wilt from invading phenolic substances, lignin and other end products all of which play vital roles in plant defense systems (Vilhena et al., 2020; Jia et al., 2023), Ordonez-Santos et al. reported that 4CL catalyzed the reaction of cinnamic acid and its hydroxyl or methoxy derivatives to obtain a coenzyme A ester, which would participate in the synthesis pathway of phenylpropane derivatives and eventually generate lignin monomers (Ordonez-Santos and Garzon-Garcia, 2021). In this study, the upregulated expression of HCT and PAL led to the accumulation of lignin to resist the pathogen invasion, and the results of this study are consistent with previous findings.

“After pathogen attack, disease-resistant banana plants initially activate the phenylpropane biosynthesis pathway, which rapidly and efficiently synthesizes lignin and flavonoids. Peroxidase is also upregulated in this pathway, enhancing the activity of key rate-limiting enzymes. In infected *Musa paradisiaca L*., the plant hormone signal transduction pathway was the main enriched pathway. The plant hormones synthesized by this pathway usually act as signaling factors to activate or regulate the expression of related disease resistance genes, but most hormones do not directly defend against pathogens. In contrast, the resistance mechanism of disease-resistant banana can be more direct and effective in counteracting damage caused by pathogenic bacteria.

Recent studies have reported on the synthesis of *C3H* and its relationship with plant lignin. Schoch et al. (2001) extracted and obtained the protein CYP98A3 from *Arabidopsis Thaliana*, which was speculated to be the *C3H*. Coleman et al. (2008) used RNA interference technology to interfere with the expression of *C3H* in hybrid poplars. After interference, the synthesis of G lignin significantly decreased, while the synthesis of H lignin increased, but the total lignin content decreased significantly. In this study, two *C3H* radicals that participate in lignin synthesis were identified from banana. The qRT−PCR ratio was employed to compare the expression of *MaC3H* in different banana varieties. The results showed that the expression of *MaC3H* was higher in all banana varieties after FocTR4 inoculation,and the amount of *C3H* in resistant varieties was greater than that in susceptible varieties. Additionally, we used Illumina HiSeq 2500 sequencing technology to analyze the transcriptomes of two *Musa paradisiaca* L. varieties. Uninfected varieties were used as control to investigate the response of *Musa paradisiaca L.* to *F. oxysporum* infection over a period of four days. Most resistance genes exhibited peak expression at 24 h post infection., and the number of differentially expressed genes (DEGs) peaked at 48 hours in the infected varieties. Notably, 46 DEGs were enriched in the plant hormone signaling pathway, suggesting genes potentially involved in blight resistance. This led to an exploration of the molecular mechanisms underlying the differences in resistance between the two varieties, representing an improvement over previous transcriptome sequencing studies.

## 5 Conclusion

Overall, the differentially expressed genes of the two varieties were enriched mainly in biological processes. The KEGG pathway analysis revealed that the phenylalanine pathway was highly expressed, where phenylalanine serves as the front reaction of lignin synthesis. Subsequently, through transcriptome data expression analysis, 14 genes related to lignin and the cell wall were selected, among which Macma4_02_g07840 (COMT) and Macma4_10_g06530 (CCOAOMT) Subsequently, relatively high expression levels. It is speculated that the varieties resistant to Fusarium wilt infection may inhibit the growth of pathogenic bacteria by restricting the synthesis of cell metabolites. This study selected two varieties of *Musa paradisiaca L.*, which are closely related but have large differences in their ability to resist blight, In this study, we used these varieties to explore the key resistance genes and main enrichment pathways more effectively, thus providing a certain theoretical reference for the genetic breeding of banana disease resistance in banana.

## Data Availability

The raw data supporting the conclusions of this article will be made available by the authors, without undue reservation.
